# From cells to insights: the power of human pluripotent stem cell-derived cortical interneurons in psychiatric disorder modeling

**DOI:** 10.3389/fpsyt.2023.1336085

**Published:** 2023-12-21

**Authors:** Peiyan Ni, Lingyi Fan, Youhui Jiang, Chuqing Zhou, Sangmi Chung

**Affiliations:** ^1^Department of Neurobiology, Affiliated Mental Health Center and Hangzhou Seventh People's Hospital, Zhejiang University School of Medicine, Hangzhou, China; ^2^Liangzhu Laboratory, State Key Laboratory of Brain-Machine Intelligence, MOE Frontier Science Center for Brain Science and Brain-Machine Integration, Zhejiang University, Hangzhou, China; ^3^NHC and CAMS Key Laboratory of Medical Neurobiology, Zhejiang University, Hangzhou, China; ^4^The Mental Health Center and Psychiatric Laboratory, West China Hospital, Sichuan University, Chengdu, China; ^5^Department of Cell Biology and Anatomy, New York Medical College, Valhalla, NY, United States

**Keywords:** interneurons, induced pluripotent stem cells, signal transduction, gene editing, GABAergic neurons, neurodevelopmental disorders, mental disorders, *in vitro* techniques

## Abstract

Psychiatric disorders, such as schizophrenia (SCZ) and autism spectrum disorders (ASD), represent a global health challenge with their poorly understood and complex etiologies. Cortical interneurons (cINs) are the primary inhibitory neurons in the cortex and their subtypes, especially those that are generated from the medial ganglionic emission (MGE) region, have been shown to play an important role in the pathogenesis of these psychiatric disorders. Recent advances in induced pluripotent stem cell (iPSC) technologies provide exciting opportunities to model and study these disorders using human iPSC-derived cINs. In this review, we present a comprehensive overview of various methods employed to generate MGE-type cINs from human iPSCs, which are mainly categorized into induction by signaling molecules vs. direct genetic manipulation. We discuss their advantages, limitations, and potential applications in psychiatric disorder modeling to aid researchers in choosing the appropriate methods based on their research goals. We also provide examples of how these methods have been applied to study the pathogenesis of psychiatric disorders. In addition, we discuss ongoing challenges and future directions in the field. Overall, iPSC-derived cINs provide a powerful tool to model the developmental pathogenesis of psychiatric disorders, thus aiding in uncovering disease mechanisms and potential therapeutic targets. This review article will provide valuable resources for researchers seeking to navigate the complexities of cIN generation methods and their applications in the study of psychiatric disorders.

## 1 Introduction

Cortical interneurons (cINs), which comprise only about 20–30% of cortical neurons, play critical roles in fine-tuning brain circuit functions; thus, their abnormalities, especially those of medial ganglionic eminence (MGE)-derived cINs, have been associated with various neurodevelopmental psychiatric disorders, such as schizophrenia (SCZ) and autism spectrum disorders (ASD). Proper interactions between inhibitory cINs and excitatory glutamatergic neurons are required to maintain a normal excitation-inhibition (E-I) balance in cortical circuits. Thus, disrupted E-I balances in cortex results in dysfunctional synchronization of neural network oscillations (1, 2), which is thought to underlie the neurodevelopmental pathogenesis of SCZ and ASD (3). However, research on these neurodevelopmental psychiatric disorders has been hampered by a lack of proper model systems that recapitulate patients’ neurodevelopmental process and disease progression since once the patients exhibit the clinical symptoms, it is impossible to go back in time to learn how neurodevelopmental dysregulation led to the pathogenesis of psychiatric disorders. Furthermore, non-neural tissue samples such as peripheral blood mononuclear cells (PBMCs) from patients are less likely to reflect functional deficits in the brain (4). Additionally, postmortem brain samples can reveal full-blown disease phenotypes but not necessarily their pathogenesis mechanisms during neurodevelopment, and moreover, are confounded by factors such as medication history and postmortem interval, etc. (5). Animal models have inherent limitations due to species differences that hinder faithful modeling of human psychiatric pathogenesis (6, 7). Considering that the divergence between human and rodent brains has caused many central nervous system therapeutics validated in rodent models to fail (8–10), it is imperative to study human developmental neurons to understand the pathogenesis mechanisms of cIN-associated neurodevelopmental psychiatric disorders.

The ability to study critical pathogenesis events in the human developmental nervous system once seemed unimaginable due to insurmountable ethical and technical barriers. However, human induced pluripotent stem cell (iPSC) technologies provide a powerful tool to study neurodevelopmental dysregulation by allowing for the generation of subject-specific and disease-relevant developmental brain cells with the same genetic makeup as subject brains in unlimited quantities, thus providing exciting opportunities to model and study neurodevelopmental disorders. Especially, several methods have been reported for the generation of cINs from iPSCs, which would be of utmost interest to scientists who study cIN-associated neurodevelopmental psychiatric disorders. Therefore, in this minireview, we will discuss different methods of cIN generation with their pros and cons, aiming to guide researchers with different questions and demands to select appropriate methods.

## 2 Generation of cINs from human pluripotent stem cells

The approaches to generate cINs from human pluripotent stem cells can be broadly divided into two major categories: the first method is cIN phenotype induction by signaling molecules to mimic natural developmental process (11) and the second method is direct genetic manipulation, which greatly shortens the differentiation timeline (12–14).

### 2.1 cIN induction by signaling molecules

One of the major ways to generate cINs from human pluripotent stem cells is by providing signaling molecules during differentiation to mimic the signaling regulations that occur during normal neurodevelopment. This method well recapitulates authentic developmental processes, compared to the transcription factor-induction method that bypasses normal developmental processes. Cells can be differentiated in two dimensions (2D) as monolayer cultures or in three dimensions (3D) as spheres. Regardless of this 2D or 3D culture format, cells are treated to enrich neuroectodermal differentiation by blocking mesoderm and endoderm differentiation with dual SMAD (BMP/TGF-β pathway inhibitors) inhibitors [BMPRIA-Fc (15), Dorsomorphin (16, 17), LDN193189 (18–22), Noggin (23), or SB431542 (15–22, 24)]. Generated neuroepithelial cells can be patterned to become specific neural cells of interest. To inhibit the induction of caudal neuroepithelium phenotypes (25), neuroepithelial cells are also treated with WNT pathway inhibitors [XAV939 (20, 22), DKK1 (15), or IWP2 (16–18, 21, 26)]. Also, these rostral neuroepithelial cells are further ventralized into ganglionic eminence (GE) cell types by sonic hedgehog pathway activators [SHH (19, 20, 22–24), Purmorphamine (15, 19, 20, 22, 27, 28), or SAG (16–18, 21, 26)]. The time window of SHH treatment impacts the phenotype induction, where one group reported generation of diencephalic phenotypes with early SHH activation (20), whereas others observed MGE-phenotype induction with early SHH activation (15, 18). It is not clear what caused such discrepancies, but there was a difference in the reagents utilized for ventralization (SHH, Purmorphamine, and SAG, etc.) with different doses, along with different base media, among these protocols; thus, side by side experiments down the road could help resolve these discrepancies. As a further improvement, some protocols include FGF8 treatment to further rostralize ventral neuroepithelium into medial ganglionic eminence (MGE) phenotypes at the expense of caudal ganglionic eminence (CGE) phenotypes (18, 21), ensuring consistent MGE induction regardless of the endogenous expression level of FGF8. Overall, based on the knowledge gained on the human neurodevelopmental process, the induction of MGE-type cINs is mainly achieved by early inhibition of SMAD and WNT signaling pathways, along with the activation of the SHH signaling pathway, providing a replicable model system for the study of the neurodevelopmental pathogenetic mechanisms. The cINs induced by signaling molecules have been successfully utilized to study the mechanisms of neurodevelopmental disorders. For example, when generated developmental cINs were co-cultured with activated microglia to mimic increased developmental risks by prenatal immune activation, Park et al., found that the SCZ patient-derived cINs presented disrupted metabolic pathways and impaired mitochondrial function, arborization, synapse formation, and synaptic GABA release, which persist in SCZ cINs even after the removal of activated microglia co-culture, but not in healthy control cINs, pointing to gene×environment interactions (29).

### 2.2 Direct genetic manipulation

Compared to the signaling molecule-based phenotype induction method, the direct genetic manipulation method, which employs exogenous expression of transcription factors that play crucial roles in cIN differentiation, can greatly the shorten phenotype induction timeline. Taking advantage of the knowledge of the transcriptional controls during cIN differentiation, cIN-inducing candidate transcription factors were screened for their ability to induce cINs (12–14, 30). Achaete-scute complex-like homolog 1 (*ASCL1*, also known as *MASH1*), which is widely expressed in the embryonic ventral brain and plays a dominant role in determining cIN identity (31–33), was able to trigger the conversion of mouse embryonic fibroblasts into tubulin beta III (TUBB3)^+^ neuronal cells on its own (30). Furthermore, overexpression of a phospho-mutant form of ASCL1 (five serine residues substituted with alanine, denoted A^SA^), with their enhanced ability to drive neuronal differentiation (34), resulted in a production of ~2-fold more microtubule-associated protein (MAP2)^+^ neurons (12). Distal-less homeobox (DLX) family members are multifunctional transcription factors that promote the differentiation of progenitors into cINs and inhibit their differentiation into glial cells (35, 36). When *DLX2* was expressed in conjunction with *ASCL1*, the iPSC-derived neurons were conferred with a cIN fate (12, 13). Likewise, *DLX5/DLX6* is also found to promote cIN generation (30). LIM homeobox 6 (*LHX6*) is a direct target of Nkx2.1, a MGE progenitor-specific transcription factor (37, 38), and favors the fate-specification of MGE precursors into parvalbumin (PV)- or somatostatin (SST)-expressing cINs (12, 14). Combining signaling molecule activation and genetic modification together; Yuan et al. showed that the SHH pathway activation along with the LHX6 induction significantly increased the percentage of PV and SST subtypes compared to the condition without *LHX6* overexpression (14). MicroRNA has also been found to benefit the neuronal conversion (39), and Sun et al., reported that the addition of miR-9/9^*^-124 into the iPSCs with *ASCL1*, *LHX6*, and *DLX2* overexpression significantly increased the percentage of MAP2^+^ cells while maintaining the high GABA^+^/MAP2^+^ ratio and enhancing dendritic arborization (12). Overall, the rapid induction of MGE-derived cINs from iPSCs can be achieved by the overexpression of transcription factors that can induce their fate determination or maturation, either on their own or supplemented by other signaling molecules. By utilizing direct genetic manipulation, Ishii et al., successfully generated cINs and glutamatergic neurons from iPSCs derived from patients with bipolar disorder (with copy number variations of PCDH15) and SCZ (with copy number variations of RELN) (40), and observed specific abnormalities in the neuronal routing and synaptic function underlying the psychiatric disorders.

### 2.3 Considerations in cIN induction method selection

For functional studies of mature cINs *in vitro*, direct genetic manipulation offer specific advantages. Ishii et al. conducted a study where they differentiated GABAergic and glutamatergic mature neurons from iPSCs obtained from bipolar disorder and SCZ patients with copy number variations of *PCDH15* and *RELN* (40). Notably, the authors compared two methods and found that direct genetic manipulation was more efficient and resulted in higher neuron maturity, enabling them to study neuronal routing and synaptic abnormality phenotypes in psychiatric disorders. The researchers in the first example investigated the developmental status of cINs to examine the correlation between prenatal immune activation and SCZ risk. In the second example, the researchers compared the mature functions of cINs with the findings of postmortem brain studies, aiming to identify shared neuronal defects across psychiatric disorders.

Depending on their specific requirements and applications, researchers can choose optimal methods of cIN generation, considering their advantages and disadvantages. The most significant difference between the two methods is the time consumption. Direct genetic regulation can obtain functional cINs in a relatively short period, which is of great use for fast screening to quickly identify potential therapeutic targets. Furthermore, direct genetic manipulation methods can also transdifferentiate terminally differentiated somatic cells (e.g., fibroblasts) directly into cINs (30), which greatly expands the application of this method, especially providing the possibility of generating neuronal populations with an aging signature (41), which is not easy to achieve in the case of iPSC-derived fetal neurons. However, caution needs to be taken, considering the fact that direct genetic modification methods bypass normal neurodevelopmental processes and may not be fully identical to their *in vivo* counterparts. In addition, the introduction of exogenous factors by viral vectors’ integration into the genome could generate potential confounders during analysis (42), especially when studying genetic risks spread throughout genome as in the case of SCZ.

In contrast, the signaling molecules induction methods require a longer time but closely recapitulate the native neurodevelopmental process, and thus are more suitable to observe and study developmental pathogenetic mechanisms (43, 44). Signaling molecule induction can be combined with self-assembled 3D neuro-organoids differentiation for the generation of more homogeneous cell population within the organoid. Signaling molecule-induced neuro-organoids exhibit cyto-architectural features of developing brains and provide a model where neuronal migration, projections, and circuit formation can be studied in 3D (17, 22, 26). Furthermore, cIN phenotype-induced neuro-organoids have been optimized for large scale spinner culture systems, allowing industrial-scale culture for the use for high throughput drug screening or cell therapy (21).

When choosing the appropriate cIN differentiation methods, it is also necessary to consider the appropriate developmental stage and maturity of cINs depending on the experimental purpose. One needs to keep in mind the fact that signaling molecule induction methods well recapitulate normal neurodevelopmental timeline, especially developmentally protracted maturation of cINs (15, 45), so the researcher needs to determine length of differentiation based on the developmental timeline of interest where specific disease pathogenesis may occur. Overall, signaling molecule-mediated induction methods can be mostly useful to model fetal neurodevelopmental processes. There has been some methods that have shown to facilitate the maturation of human pluripotent stem cell-derived neurons such as co-culture with astrocytes (12, 13, 15, 30), co-culture with glutamatergic neurons (20), and grafting them into the animal brains (14). Still, these methods have not allowed for generation of fully mature fast-spiking cINs like in adult brains. Alternatively, disease modeling for more mature stages of neurons may be accomplished by utilizing genetic modification along with the use of more mature somatic cells as a starting material (41). In case cINs are studied after transplantation into rodent brains, it would be prudent to use post-mitotic neurons to avoid the potential of uncontrolled proliferation that can damage host cyto-architecture. Several reagents have been proven to accelerate cell cycle exit without affecting cIN phenotype, including CultureOne (Invitrogen), NOTCH pathway inhibitors (DAPT, 2634 Tocris), MEK inhibitors (PD0325901 and 4,192 Tocris), and CDK pathway inhibitors (PD0332991 and S1116 Selleck Chemicals) (21, 46). Use of synchronized cIN populations by induced cell cycle exit will be also useful to obtain reliable disease modeling results by avoiding heterogenous maturity within generated cell populations that can confound the assay results.

Based on molecular markers and the expression of neuropeptides or calcium-binding proteins, cINs can be divided into many subtypes such as somatostatin (SST)^+^, parvalbumin (PV)^+^, calbindin (CB)^+^, calretinin (CR)^+^, neuronal nitric oxide synthase (nNOS), and vasoactive intestinal peptide (VIP)^+^ cINs. The MGE progenitors give rise mostly to SST^+^ and PV^+^ interneurons (47), and among these, PV neurons requires an lengthy time of maturation that matches their protracted maturation during *in vivo* development (15, 20). Among various subtypes, CB^+^, CR^+^, and SST^+^ cINs start to appear at early differentiation stage during MGE-type cIN generation and their populations increase over time, whereas PV^+^, nNOS^+^, and VIP^+^ neurons can be observed at a very low proportion even after several months’ differentiation (15–17, 20, 27). Hence, one needs to be aware of the changing composition of cIN subtypes as the maturation process goes on, and the timeline of assays may need to be adjusted depending on the cINs subtype of interest.

The cIN induction methods reviewed in this review (Table 1) provide relatively homogeneous cell populations, which are useful for genomics studies without the confounders resulting from changing and heterogeneous cell populations. Such homogeneity can provide advantages in the study of cellular and molecular mechanisms of neurodevelopmental disorders (9, 29, 48–50). However, such homogeneous populations lack interactions among diverse cell types as in the brain. Un-induced organoids or organoids assembled after induction can compensate for such a limitation, providing more physiological cell–cell interactions, and cytoarchitectures (16, 26, 51–53), and even circuit-level functionalities *in vitro* or *in vivo* (54–57).

**Table 1:** The cIN induction methods reviewed in this review.

Methods	References	Timeline	Medium	Adherent/suspension	Patterning factors (signaling molecules)/infected TFs	cIN phenotypes and functions
Signaling molecules induction	Maroof et al. (20)	D0-D10: Neuroepithelium induction	85% DMEM, 15% KSR, 2 mM L-Glut, and 10 μM 2-ME KSR is gradually shifted to N2: from day 5, increasing every 2 days (25, 50, 75%)	Adherent	LDN193189: 100 nM SB431542: 10 μM XAV939: 2 μM	D18: ~60% Nkx2.1^+^; D32: ~40% nNOS, ~80% Cal, ~40% SST, and ~ 40% PV of Nkx2.1-GFP^+^ cells; D60: ~80% are GABA^+^ cells; Cells receive excitatory and inhibitory synaptic inputs
		D10-D18: Ventral patterning	Neurobasal media, B27, 2 mM L-Glut, 10 ng/mL BDNF, 200 μM AA, and 200 μM Dibutyryl-cAMP	Adherent	Purmorphamine: 1–2 μM SHH: 5 nM	
		D18-: Maturation and maintenance	Neurobasal media, B27, 10 ng/mL BDNF, 200 μM AA, and 200 μM Dibutyryl-cAMP Coculture with mouse cortical neurons from D30	Adherent	NA	
	Germain et al. (23)	D0-D6: Neuroepithelium generation	Neurobasal medium with N2, B27, insulin-transferrin-selenium, and L-Glut	Adherent	Noggin: 500 ng/mL	D17: 46.7% NKX2.1^+^ cells by FACS
		D6-D17: Ventral patterning		Adherent	rhSHH-N: 500 ng/mL	
		D17-D35: Mature rosettes generation		Adherent	NA	
		D35-: NPCs generation		Suspension		
	Nicholas et al. (15)	D0-D14: Ventral forebrain Patterning	Neurobasal-A, 2% B27-Vitamin A, 20% KSR, 1% NEAA, and 440 nM 2-ME	D0-D7: Suspension D7-D14: Adherent	SB431542: 10 μM BMPRIA-Fc: 1.5 μg/mL DKK1: 1 μg/mL Purmorphamine: 1–2 μM	D20–30: >90% NKX2.1^+^; D35: ~90% FOXG1^+^, ~90% ASCL1^+^, ~95% TUJ^+^, ~85% GABA^+^, and ~ 90%^+^ of Nkx2.1-GFP^+^ cells; W30: maturation of AP firing properties
		D14-: Maturation and maintenance	Neurobasal-A, 2% B27-Vitamin A, 20% KSR, 1% NEAA, and 440 nM 2-ME Coculture with mouse glia	Adherent	D14-D35: Purmorphamine: 1 μM D35-: NA	
	Liu et al. (27) and Yuan et al. (28)	D0-D9: Neuroepithelium induction	D0-D1: 78% DMEM/F12, 20% KSR, 4 ng/mL FGF2, 1% NEAA, 2 mM GlutaMAX, 396 nM 2-ME; D1-D4: 78% DMEM/F12, 20% KSR, 1% NEAA, 1% 100× GlutaMAX, 396 nM 2-ME; D4-D9: 98% DMEM/F12, 1% NEAA, 1% N2, and 2 μg/mL of heparin	D0-D6: Suspension D7-D9: Adherent	NA	D10: >90% PAX6^+^ neuroepithelial cells; D20-25: ~90% NKX2.1^+^; D45: > 90% of neurons are GABA^+^; Na and K channels are mature and result in the AP firing upon current injection; a functional synaptic network is formed with surrounding neurons
		D10-D25: Ventral patterning	D10-D16: 98% DMEM/F12, 1% NEAA, 1% N2, and 2 μg/mL of heparin; D16-D25: 96% DMEM/F12, 2% B27, 1% NEAA, 1% N2, and 2 μg/mL of heparin	D10-D16: Adherent D16-D25: Suspension	SHH (C24II): 1 mg/mL, SHH (C25II): 300 ng/ML, or Purmorphamine: 1.5 μM	
		D25-: maturation and maintenance	98% neurobasal, 1% NEAA, 1% N2, 1 μM cAMP, 10 ng/mL BDNF, 10 ng/mL GDNF, and 10 ng/mL IGF1	Adherent	NA	
	Kim et al. (18) and Ni et al. (21)	D0-D21: MGE phenotype induction	D0-D7: DMEM-GlutaMAX, 15% KSR, and 55 μM 2-ME	Suspension	LDN193189: 100 nM SB431542: 10 μM SAG: 100 nM IWP2: 5 μM	D21: ~70% cells are NKX2.1^+^; D42: >95% cells are SOX6^+^, GAD1^+^, and β-III TUB^+^; W12: a majority of cINs fired AP
			D7-D14: DMEM/F12-GlutaMAX, 15% KSR, and 55 μM 2-ME	Suspension	LDN193189: 100 nM SAG: 100 nM	
			D14-D21: DMEM/F12-GlutaMAX, 1% N2, and 10 μg/mL AA	Suspension	SAG: 100 nM FGF8: 50 ng/mL	
		D21-: maturation and maintenance	D21-D28: DMEM/F12-GlutaMAX, 1% N2, 10 μg/mL AA, 5 ng/mL BDNF, and 5 ng/mL GDNF	Suspension	NA	
			D28-: DMEM/F12-GlutaMAX, 2% B27, 5 ng/mL BDNF, and 5 ng/mL GDNF	Suspension	NA	
	Birey et al. (16) and Sloan et al. (17)	D0-D6: Neuroepithelium induction	DMEM/12, 20% KSR, 1% NEAA, 1 mM GlutaMAX, and 0.1 mM 2-ME	Suspension	D0-D4: Dorsomorphin: 5 μM; SB431532: 10 μM D4-D6: Dorsomorphin: 5 μM; SB431532: 10 μM; IWP-2: 5 μM	D25: ~70% NKX2.1; D60: ~30% GABA and GAD67 with GABAergic subtype marker expression (SST, CR, and CB); ~75% of neurons generated AP in response to depolarization; around 60% of neurons exhibit spontaneous IPSCs
		D6-D43: Ventral forebrain patterning	D6-D25: Neurobasal A medium, 2% B27, 2 mM GlutaMAX, 20 ng/mL EGF, and 20 ng/mL FGF2	Suspension	D6-D24: IWP-2: 5 μM D12-D24: SAG: 100 nM D12-D15: RA: 100 nM D15-D24:AlloP: 100 nM	
			D25-D43: Neurobasal medium, 2% B27, 2 mM GlutaMAX, 20 ng/mL BDNF, and 20 ng/mL NT3	Suspension	NA	
		D43-: Maturation and maintenance	Neurobasal medium, 2% B27, and 2 mM GlutaMAX	Suspension	NA		
	Xiang et al. (22)	D0-D10: Neuroepithelium induction	DMEM/F12, 15% KSR, 1% MEM-NEAA, 2 mM Glutamax, 100 μM 2-ME, 50 μM Y-27632 (D0-D4), and 5% FBS (only D1)	Suspension	LDN193189: 100 nM SB431542: 10 μM XAV939: 2 μM	D21: 82.40% cells express NKX2.1; 93.97% cells are FOXG1^+^
		D10-D18: Ventral patterning	DMEM/F12, 1.5 mg/mL Detrose, 100 μM 2-ME, 1% N2, and 2% B27-vitamin A	Suspension	rhSHH: 100 ng/mL Purmorphamine: 1 μM	
		D18-: maturation and maintenance	DMEM/F12 media: Neurobasal media = 1:1, supplemented with 0.5% N2, 1% B27-Vitamin A, 2 mM Glutamx, 0.5% MEM-NEAA, 0.025% (v/v) human insulin, 50 μM 2-ME, 20 ng/mL BDNF, 200 mM cAMP, and 200 μM AA	Suspension	NA	
	Bagley et al. (26)	D0-D5: Neuroepithelium induction	D0-D3: DMEM-F12, 20% KSR, 3% FBS, 2 mM GlutaMAX, 1% MEM-NEAA, 385 nM 2-ME, 4 ng/mL FGF2, and 50 μM Y27632 D3-D5: DMEM-F12, 20% KSR, 3% FBS, 2 mM GlutaMAX, 1% MEM-NEAA, 385 nM 2-ME	Suspension	NA	D80: the cINs can migrate within fused ventral::dorsal organoids; ~40% of the migrating GFP^+^/GAD1^+^ cells are SOX6^+^, 6% SST^+^, 6% NPY^+^, 20% CB^+^, and 5% PV^+^
		D5-D12: Ventral forebrain patterning	DMEM-F12, 1% N2, 2 mM GlutaMAX, 1% MEM-NEAA, and 1 μg/mL heparin.	D5-D11: Suspension D12: Embedding in Matrigel droplets	D5-D11: IWP2: 2.5 μΜ SAG: 100 nM	
		D12-: Maturation and maintenance	DMEM-F12 media:Neurobasal media = 1:1, 0.5% N2, 1% B27 (D12-D16: without Vitamin A, D16-: with Vitamin A), 192.6 nM 2-ME, 2 mM GlutaMAX, 0.5% MEM-NEAA, and 2.5 μg/mL insulin	Suspension	NA	
Direct genetic reprogramming	Colasante et al. (30)	D1-: Lentiviral infection	mTeSR1 media	Adherent	*ASCL1*, *DLX5*, *DLX6*, *FOXG1*, and *SOX2*	D21: more than 50% of MAP2^+^ cells are GABA^+^; 90% co-express PV, while only occasionally positive for SST; more than 30% of the total cells were estimated to be GABAergic neurons; D36: Na^+^ and K^+^ currents, fired repetitive APs, and spontaneous GABAergic synaptic activity	
		D0-D12: cIN induction	DMEM/F12, N2, 1% NEAA, 10 ng/mL BDNF, 0.2 μg/mL laminin, and 2 μg/mL DOX Co-culture with mouse hippocampal primary neurons from D8	Adherent			
		D12-: Maturation and maintenance	Neurobasal medium, 2 mM glutamine, B27, 10 ng/mL BDNF and addition of Ara-C (5 μM from D12-14)	Adherent			
	Sun et al. (12)	D0: Lentiviral infection	mTeSR1 media, 1 μM thiazovivin.	Adherent	*ASCL1^SA^*, *LHX6*, and *DLX2* and miR-9/9*-124	D42: 84.5% of MAP2^+^ cells are GABA^+^; D42–D56: subtypes of cINs including SST (24.3%), CR (11.6%), CB (6.5%), and NPY (5.4%); maturing K^+^ and Na^+^ currents; different types of AP firing patterns similar to cINs; functional postsynaptic machinery, and reception of inhibitory and excitatory synaptic inputs; D70-: ~1% PV+ neurons	
		D1-D10: cIN induction	ScienCell Neuronal Medium, blasticidin (D3-D7), puromycin (D3-D7), and 1 μg/mL DOX (D3-D10)	Adherent D7: dissociation			
		D10-: Maturation and maintenance	ScienCell Neuronal Medium, 10 ng/mL BDNF, 10 ng/mL GDNF, 10 ng/mL NT3, 10 ng/mL, and IGF1 1 μg/mL DOX (D10-D21) Co-cultured with primary rat glia since D14-D20	Adherent			
	Yang et al. (13)	D1-: Lentivirus infection	mTeSR1 media with 2 μM thiazovivin	Adherent	*ASCL1* and *DLX2*	W5: almost all neurons expressed the forebrain marker FOXG1 (93.5%) and GABAergic neuron markers such as GABA (89.1%), DLXs (88.5%), and GAD65/67 (94.4%); W7: a progressive maturation of spontaneous and evoked IPSCs, and excitatory synaptic inputs can be received after being cocultured with glutamatergic cells since D10	
		D0-D7: cIN induction	DMEM/F12, N2, 1% NEAA, 2 g/L DOX, hygromycin (D1-D3), and puromycin (D1-D3)	Adherent D6: Dissociation and co-culture with mouse-glial-cells			
		D7-: Maturation and maintenance	Neurobasal medium, B27, Glutamax, 20 ng/mL BDNF, and 2 g/L Ara-C	Adherent Co-culture with mouse-glial-cells			
	Yuan et al. (14)	D0: Lentivirus infection	E8 medium	Adherent	*LHX6* 500 nM SAG (D10-D25)	D25: more than 80% of the cells showed expression of NKX2.1; D85: SST^+^ neurons increased to 29% and PV^+^ neurons to 21%	
		D0-D25: cIN induction	D-D1: 50% E8, 49% DMEM/F12, 0.5% NEAA, 0.5% N2, and 1 μg/mL of heparin; D1-D10: 98% DMEM/F12, 1% NEAA, 1% N2, and 2 μg/mL heparin D10-D16: 98% DMEM/F12, 1% NEAA, 1% N2, 2 μg/mL of heparin, and 3 μg/mL DOX; D16-D25: 96% DMEM/F12, 2% B27, 1% NEAA, 1% N2, 2 μg/mL of heparin, and 3 μg/mL DOX	D0-D7: Suspension D7-D10: Adherent D10-D16: Adherent D16-D25: Suspension			
		D25-: Maturation and maintenance	NDM medium: 98% neurobasal, 1% NEAA, 1% N2, 1 μM cAMP, 10 ng/mL BDNF, 10 ng/mL GDNF, and 10 ng/mL IGF1	Adherent		

2-ME, 2-Mercaptoethanol, reducing agent; AA, Ascorbic acid, antioxidant; AHP, Afterhyperpolarization; AlloP, Allopregnanolone; AP, Action potential; Ara-C, Cytarabine; ASCL1, Achaete-scute homolog 1; B27, B27 supplement; BDNF, Brain-derived neurotrophic factor; BMPRIA-Fc, Bone morphogenetic protein receptor 1a Fc chimera, BMP pathway inhibitor; CB, Calbindin; CR, Calcium-binding protein; Cm, Membrane capacitance; dbcAMP, dibutyryl-cyclic AMP, cAMP pathway activator; DCX, Doublecortin; DKK1, Dickkopf homolog 1, Wnt pathway inhibitor; DLX2, Homeobox protein distal-less homeobox 2; DMEM/F12, Dulbecco’s modified Eagle medium nutrient mixture F-12; DOX, Doxycycline; EB, Embryoid body; EGF, Epidermal growth factor; EMX1, Empty spiracles homeobox 1; FACS, Fluorescence-activated Cell Sorting; FBS, Fetal bovine serum; FGF2, Basic fibroblast growth factor; FGF8, Fibroblast growth factor 8; FOXG1, Forkhead box protein G1; GAD1, Glutamate decarboxylase 1; GAD65, Glutamate decarboxylase 65; GAD67, Glutamate decarboxylase 67; GDNF, Glial cell line-derived neurotrophic factor; hPSCM, human PSC medium; IGF1, Insulin-like growth factor; ITS-G, insulin-transferrin-selenium; IWP2, Wnt pathway inhibitor; KSR, Knockout serum replacement; LDN193189, BMP pathway inhibitor; L-glut, L-glutamine; LHX6, LIM/homeobox protein 6; MAP2, Microtubule-associated protein 2; N2, N2 supplement; NDM, Neural differentiation medium; NEAA, Nonessential amino acids; Nestin, Neuroepithelial stem cell protein; NeuN, Hexaribonucleotide binding protein-3; NIM, Neural induction medium; NKX2.1, NK2 homeobox 1, also known as thyroid transcription factor 1 (TTF-1); Noggin, BMP pathway inhibitor; NPC, Neural progenitor cell; NPY, Neuropeptide Y; NT3, Neurotrophin3, neurotrophic factor; RA, Retinoic acid; PAX6, Paired box protein Pax-6; RMP, Restive membrane potential; rhSHH-N, recombinant human SHH with an N-terminus modification; RELN, Reelin; Rm, Membrane resistance; SAG, Hedgehog pathway activator; SB431542, BMP/TGF-β pathway inhibitor; SHH, Sonic Hedgehog, developmental morphogen; TUJ1, Class III β-tubulin; vGAT, Vesicular GABA transporter; XAV939, WNT pathway inhibitor; Y27632, Rho-associated kinase (ROCK) pathway inhibitor.

### 2.4 Future improvements

To date, iPSC-derived cINs have been increasingly used to study the pathogenesis mechanisms of neurodevelopmental disorders and have shown great potential. However, there are still issues that need to be addressed for the realization of their full potential. Long-term culture processes are needed to obtain more mature cINs, and in general, neuro-organoid cultures are more robust for this purpose than adherent cultures. Still, there are issues with neuro-organoid long-term culture, including insufficient nutrient delivery in large spheres and difficulty with structural maintenance. Possible solutions for these issues are: (1) biocompatible 3D scaffolds that can provide appropriate mechanical support, and whose spatial structure can also provide channels to transport nutrients and oxygen to the cells in the inner layer of the sphere. Such approaches lead to the attenuation of the hypoxic response pathway, lower metabolic dysfunction, and decreased interior cell death (58). (2) vascularization that can create a more physiological microenvironment support for 3D neuro-organoids (59–61). Several methods have been tested for this approach, including co-culturing the neurospheres with endothelial cells (ECs) differentiated from iPSCs (62), or co-culturing iPSCs with human umbilical vein endothelial cells (HUVECs) (63). The vascularized organoids exhibited robust neurogenesis and chemical and electrical synapses *in vitro*, as well as constructed functional blood vessels inside the grafts and in-between human-mouse interfaces after transplantation *in vivo*. (3) “trimming” the organoid into slices and exposing the interior of organoids to the culture environment leads to sustained neurogenesis, which also bypasses the diffusion limit to prevent cell death over long-term cultures. This method leads to sustained neurogenesis and the formation of an expanded cortical plate which resembles late-stage cortical development (64).

As mentioned above, the development of fast-spiking PV interneurons and their circuit connectivity is quite challenging to achieve during *in vitro* differentiation, because of their prolonged maturation process that recapitulates the *in vivo* developmental process where many of them start to express PV only post-natally and reach adolescence to complete maturation (65, 66). There have been several efforts to facilitate the generation of PV neurons from iPSCs, including the use of a potent adenylate cyclase activator Forskolin (Coleonol) (67) and overexpression of transcription factors of *ASCL1* (67) and *LHX6* (14). However, it is still a time-consuming process to generate PV interneurons from iPSCs [>2 months to generate ~20% PV^+^ cINs with the overexpression of *LHX6* together with SHH activator (14)], and fully mature fast-spiking-cINs are still difficult to achieve even after months’ culture *in vitro* or grafting *in vivo*. To compensate for a low % of PV generation at this gestational period, FACS sorting of PV^+^ neurons was attempted to enrich them utilizing cell type-specific reporter expression (68). However, achieving their full maturation to model their adult phenotype is still awaiting further technical development.

## 3 Conclusion

In this review, we discussed diverse methods of generating inhibitory cINs from iPSCs, including the advantages and disadvantages of different methods. Researchers will need to select appropriate cIN generation methods based on the scientific questions and requirements. There are still aspects of current cIN modeling that needs further improvement, such as how to better simulate physiological neurodevelopmental environments and how to generate more mature cell types beyond the fetal neural types generated in many cases. This can be the focus of future research to be optimized. In summary, we systematically reviewed the various methods of cIN generation from human pluripotent stem cells, which will provide valuable tools to study the mechanisms of neurodevelopmental disorders in real human tissues during developmental time points of their vulnerability and to develop novel therapeutics based on a human model system.

## Author contributions

SC: Supervision, Validation, Writing – original draft, Writing – review & editing. PN: Supervision, Validation, Writing – original draft, Writing – review & editing. LF: Writing – original draft, Writing – review & editing. YJ: Conceptualization, Writing – review & editing. CZ: Writing – review & editing.

## References

[1] Chattopadhyaya B Cristo GD. Gabaergic circuit dysfunctions in neurodevelopmental disorders. Front Psych. (2012) 3:51. doi: 10.3389/fpsyt.2012.00051 PMC3364508 22666213

[2] Gonzalez-Burgos G Fish KN Lewis DA. Gaba neuron alterations, cortical circuit dysfunction and cognitive deficits in schizophrenia. Neural Plast. (2011) 2011:723184. doi: 10.1155/2011/723184 21904685 PMC3167184

[3] Yang J Yang X Tang K. Interneuron development and dysfunction. FEBS J. (2021) 289:2318–36. doi: 10.1111/febs.15872 33844440

[4] Sequeira A Martin MV Rollins B Moon EA Bunney WE Macciardi F. Mitochondrial mutations and polymorphisms in psychiatric disorders. Front Genet. (2012) 3:103. doi: 10.3389/fgene.2012.00103 22723804 PMC3379031

[5] Ramaker RC Bowling KM Lasseigne BN Hagenauer MH Hardigan AA Davis NS. Post-mortem molecular profiling of three psychiatric disorders. Genome Med. (2017) 9:72. doi: 10.1186/s13073-017-0458-5, PMID: 28754123 PMC5534072

[6] Kaiser T Feng G. Modeling psychiatric disorders for developing effective treatments. Nat Med. (2015) 21:979–88. doi: 10.1038/nm.3935, PMID: 26340119 PMC4886231

[7] Salgado JV Sandner G. A critical overview of animal models of psychiatric disorders: challenges and perspectives. Rev Bras Psiquiatr. (2013) 35:S77–81. doi: 10.1590/1516-4446-2013-1156, PMID: 24271229

[8] Hodge RD Bakken TE Miller JA Smith KA Barkan ER Graybuck LT. Conserved cell types with divergent features in human versus mouse cortex. Nature. (2019) 573:61–8. doi: 10.1038/s41586-019-1506-7, PMID: 31435019 PMC6919571

[9] Shao Z Noh H Bin Kim W Ni P Nguyen C Cote SE. Dysregulated protocadherin-pathway activity as an intrinsic defect in induced pluripotent stem cell-derived cortical interneurons from subjects with schizophrenia. Nat Neurosci. (2019) 22:229–42. doi: 10.1038/s41593-018-0313-z, PMID: 30664768 PMC6373728

[10] Zhao X Bhattacharyya A. Human models are needed for studying human neurodevelopmental disorders. Am J Hum Genet. (2018) 103:829–57. doi: 10.1016/j.ajhg.2018.10.009, PMID: 30526865 PMC6288051

[11] Simão D Silva MM Terrasso AP Arez F Sousa MFQ Mehrjardi NZ. Recapitulation of human neural microenvironment signatures in IPSC-derived NPC 3D differentiation. Stem Cell Rep. (2018) 11:552–64. doi: 10.1016/j.stemcr.2018.06.020, PMID: 30057262 PMC6094163

[12] Sun AX Yuan Q Tan S Xiao Y Wang D Khoo AT. Direct induction and functional maturation of forebrain gabaergic neurons from human pluripotent stem cells. Cell Rep. (2016) 16:1942–53. doi: 10.1016/j.celrep.2016.07.035, PMID: 27498872

[13] Yang N Chanda S Marro S Ng Y-H Janas JA Haag D. Generation of pure Gabaergic neurons by transcription factor programming. Nat Methods. (2017) 14:621–8. doi: 10.1038/nmeth.4291, PMID: 28504679 PMC5567689

[14] Yuan F Chen X Fang KH Wang Y Lin M Xu SB. Induction of human somatostatin and parvalbumin neurons by expressing a single transcription factor Lim homeobox 6. elife. (2018) 7:e37382. doi: 10.7554/eLife.37382, PMID: 30251953 PMC6181563

[15] Nicholas CR Chen J Tang Y Southwell DG Chalmers N Vogt D. Functional maturation of HPSC-derived forebrain interneurons requires an extended timeline and mimics human neural development. Cell Stem Cell. (2013) 12:573–86. doi: 10.1016/j.stem.2013.04.005, PMID: 23642366 PMC3699205

[16] Birey F Andersen J Makinson CD Islam S Wei W Huber N. Assembly of functionally integrated human forebrain spheroids. Nature. (2017) 545:54–9. doi: 10.1038/nature22330, PMID: 28445465 PMC5805137

[17] Sloan SA Andersen J PaȘca AM Birey F PaȘca SP. Generation and assembly of human brain region-specific three-dimensional cultures. Nat Protoc. (2018) 13:2062–85. doi: 10.1038/s41596-018-0032-7, PMID: 30202107 PMC6597009

[18] Kim T-G Yao R Monnell T Cho J-H Vasudevan A Koh A. Efficient specification of interneurons from human pluripotent stem cells by dorsoventral and rostrocaudal modulation. Stem Cells. (2014) 32:1789–804. doi: 10.1002/stem.1704, PMID: 24648391 PMC4147720

[19] Kriks S Shim J-W Piao J Ganat YM Wakeman DR Xie Z. Dopamine neurons derived from human Es cells efficiently engraft in animal models of Parkinson’s disease. Nature. (2011) 480:547–51. doi: 10.1038/nature10648, PMID: 22056989 PMC3245796

[20] Maroof AM Keros S Tyson JA Ying SW Ganat YM Merkle FT. Directed differentiation and functional maturation of cortical interneurons from human embryonic stem cells. Cell Stem Cell. (2013) 12:559–72. doi: 10.1016/j.stem.2013.04.008, PMID: 23642365 PMC3681523

[21] Ni P Noh H Shao Z Zhu Q Guan Y Park JJ. Large-scale generation and characterization of homogeneous populations of migratory cortical interneurons from human pluripotent stem cells. Mol Ther Methods Clin Dev. (2019) 13:414–30. doi: 10.1016/j.omtm.2019.04.002, PMID: 31061832 PMC6495066

[22] Xiang Y Tanaka Y Patterson B Kang YJ Govindaiah G Roselaar N. Fusion of regionally specified HPSC-derived organoids models human brain development and interneuron migration. Cell Stem Cell. (2017) 21:383–398.e7. doi: 10.1016/j.stem.2017.07.007, PMID: 28757360 PMC5720381

[23] Germain ND Banda EC Becker S Naegele JR Grabel LB. Derivation and isolation of Nkx2.1-positive basal forebrain progenitors from human embryonic stem cells. Stem Cells Dev. (2013) 22:1477–89. doi: 10.1089/scd.2012.0264, PMID: 23351095 PMC4854221

[24] Chambers SM Fasano CA Papapetrou EP Tomishima M Sadelain M Studer L. Highly efficient neural conversion of human Es and ips cells by dual inhibition of Smad signaling. Nat Biotechnol. (2009) 27:275–80. doi: 10.1038/nbt.1529, PMID: 19252484 PMC2756723

[25] Watanabe K Kamiya D Nishiyama A Katayama T Nozaki S Kawasaki H. Directed differentiation of telencephalic precursors from embryonic stem cells. Nat Neurosci. (2005) 8:288–96. doi: 10.1038/nn1402, PMID: 15696161

[26] Bagley JA Reumann D Bian S Lévi-Strauss J Knoblich JA. Fused cerebral organoids model interactions between brain regions. Nat Methods. (2017) 14:743–51. doi: 10.1038/nmeth.4304, PMID: 28504681 PMC5540177

[27] Liu Y Liu H Sauvey C Yao L Zarnowska ED Zhang S-C. Directed differentiation of forebrain Gaba interneurons from human pluripotent stem cells. Nat Protoc. (2013) 8:1670–9. doi: 10.1038/nprot.2013.106, PMID: 23928500 PMC4121169

[28] Yuan F Fang K-H Cao S-Y Qu Z-Y Li Q Krencik R. Efficient generation of region-specific forebrain neurons from human pluripotent stem cells under highly defined condition. Sci Rep. (2015) 5:18550. doi: 10.1038/srep18550, PMID: 26670131 PMC4680876

[29] Park GH Noh H Shao Z Ni P Qin Y Liu D. Activated microglia cause metabolic disruptions in developmental cortical interneurons that persist in interneurons from individuals with schizophrenia. Nat Neurosci. (2020) 23:1352–64. doi: 10.1038/s41593-020-00724-1 33097921 PMC7769122

[30] Colasante G Lignani G Rubio A Medrihan L Yekhlef L Sessa A. Rapid conversion of fibroblasts into functional forebrain gabaergic interneurons by direct genetic reprogramming. Cell Stem Cell. (2015) 17:719–34. doi: 10.1016/j.stem.2015.09.002, PMID: 26526726

[31] Fode C Ma Q Casarosa S Ang SL Anderson DJ Guillemot F. A role for neural determination genes in specifying the dorsoventral identity of telencephalic neurons. Genes Dev. (2000) 14:67–80. doi: 10.1101/gad.14.1.67, PMID: 10640277 PMC316337

[32] Long JE Cobos I Potter GB Rubenstein JL. Dlx1&2 and Mash1 transcription factors control Mge and Cge patterning and differentiation through parallel and overlapping pathways. Cereb Cortex. (2009) 19:i96–i106. doi: 10.1093/cercor/bhp045, PMID: 19386638 PMC2693539

[33] Schuurmans C Guillemot F. Molecular mechanisms underlying cell fate specification in the developing telencephalon. Curr Opin Neurobiol. (2002) 12:26–34. doi: 10.1016/S0959-4388(02)00286-6, PMID: 11861161

[34] Ali FR Cheng K Kirwan P Metcalfe S Livesey FJ Barker RA. The phosphorylation status of Ascl1 is a key determinant of neuronal differentiation and maturation *in vivo* and *in vitro*. Development. (2014) 141:2216–24. doi: 10.1242/dev.106377 24821983

[35] Anastasiades PG Butt SJ. Decoding the transcriptional basis for Gabaergic interneuron diversity in the mouse neocortex. Eur J Neurosci. (2011) 34:1542–52. doi: 10.1111/j.1460-9568.2011.07904.x, PMID: 22103412

[36] Wonders C Anderson SA. Cortical interneurons and their origins. Neuroscientist. (2005) 11:199–205. doi: 10.1177/1073858404270968 15911869

[37] Du T Xu Q Ocbina PJ Anderson SA. Nkx2.1 specifies cortical interneuron fate by activating Lhx6. Development. (2008) 135:1559–67. doi: 10.1242/dev.015123, PMID: 18339674

[38] Neves G Shah MM Liodis P Achimastou A Denaxa M Roalfe G. The Lim homeodomain protein Lhx6 regulates maturation of interneurons and network excitability in the mammalian cortex. Cereb Cortex. (2013) 23:1811–23. doi: 10.1093/cercor/bhs159, PMID: 22710612 PMC3698365

[39] Yoo AS Sun AX Li L Shcheglovitov A Portmann T Li Y. Microrna-mediated conversion of human fibroblasts to neurons. Nature. (2011) 476:228–31. doi: 10.1038/nature10323, PMID: 21753754 PMC3348862

[40] Ishii T Ishikawa M Fujimori K Maeda T Kushima I Arioka Y. *In vitro* modeling of the bipolar disorder and schizophrenia using patient-derived induced pluripotent stem cells with copy number variations of PCDH15 and RELN. eNeuro. (2019) 6:ENEURO.0403-18.2019. doi: 10.1523/ENEURO.0403-18.2019 PMC6800292 31540999

[41] Mertens J Paquola ACM Ku M Hatch E Böhnke L Ladjevardi S. Directly reprogrammed human neurons retain aging-associated transcriptomic signatures and reveal age-related nucleocytoplasmic defects. Cell Stem Cell. (2015) 17:705–18. doi: 10.1016/j.stem.2015.09.001, PMID: 26456686 PMC5929130

[42] Fernandopulle MS Prestil R Grunseich C Wang C Gan L Ward ME. Transcription factor-mediated differentiation of human IPSCS into neurons. Curr Protoc Cell Biol. (2018) 79:e51. doi: 10.1002/cpcb.51 29924488 PMC6993937

[43] Mariani J Coppola G Zhang P Abyzov A Provini L Tomasini L. Foxg1-dependent dysregulation of gaba/glutamate neuron differentiation in autism spectrum disorders. Cells. (2015) 162:375–90. doi: 10.1016/j.cell.2015.06.034, PMID: 26186191 PMC4519016

[44] Wang P Mokhtari R Pedrosa E Kirschenbaum M Bayrak C Zheng D. Crispr/Cas9-mediated heterozygous knockout of the autism gene Chd8 and characterization of its transcriptional networks in cerebral organoids derived from IPS cells. Mol Autism. (2017) 8:11. doi: 10.1186/s13229-017-0124-1, PMID: 28321286 PMC5357816

[45] Anderson S Vanderhaeghen P. Cortical neurogenesis from pluripotent stem cells: complexity emerging from simplicity. Curr Opin Neurobiol. (2014) 27:151–7. doi: 10.1016/j.conb.2014.03.012, PMID: 24747604 PMC4386058

[46] Bershteyn M Broer S Parekh M Maury Y Havlicek S Kriks S. Human pallial Mge-type Gabaergic interneuron cell therapy for chronic focal epilepsy. Cell Stem Cell. (2023) 30:1331–1350.e11. doi: 10.1016/j.stem.2023.08.013 37802038 PMC10993865

[47] Le Magueresse C Monyer H. Gabaergic interneurons shape the functional maturation of the cortex. Neuron. (2013) 77:388–405. doi: 10.1016/j.neuron.2013.01.011, PMID: 23395369

[48] Lu K Hong Y Tao M Shen L Zheng Z Fang K. Depressive patient-derived Gaba interneurons reveal abnormal neural activity associated with Htr2C. EMBO Mol Med. (2023) 15:e16364. doi: 10.15252/emmm.202216364 36373384 PMC9832822

[49] Xu L Huo HQ Lu KQ Tang XY Hong Y Han X. Abnormal mitochondria in Down syndrome ipsc-derived Gabaergic interneurons and organoids. Biochim Biophys Acta Mol basis Dis. (2022) 1868:166388. doi: 10.1016/j.bbadis.2022.166388, PMID: 35301086

[50] Xu R Brawner AT Li S Liu JJ Kim H Xue H. Olig2 drives abnormal neurodevelopmental phenotypes in human IPSC-based organoid and chimeric mouse models of down syndrome. Cell Stem Cell. (2019) 24:908–926.e8. doi: 10.1016/j.stem.2019.04.014, PMID: 31130512 PMC6944064

[51] Kathuria A Lopez-Lengowski K Jagtap SS Mcphie D Perlis RH Cohen BM. Transcriptomic landscape and functional characterization of induced pluripotent stem cell-derived cerebral organoids in schizophrenia. JAMA Psychiatry. (2020) 77:745–54. doi: 10.1001/jamapsychiatry.2020.0196, PMID: 32186681 PMC7081156

[52] Li C Fleck JS Martins-Costa C Burkard TR Themann J Stuempflen M. Single-cell brain organoid screening identifies developmental defects in autism. Nature. (2023) 621:373–80. doi: 10.1038/s41586-023-06473-y, PMID: 37704762 PMC10499611

[53] Notaras M Lodhi A Dündar F Collier P Sayles NM Tilgner H. Schizophrenia is defined by cell-specific neuropathology and multiple neurodevelopmental mechanisms in patient-derived cerebral organoids. Mol Psychiatry. (2022) 27:1416–34. doi: 10.1038/s41380-021-01316-6, PMID: 34789849 PMC9095467

[54] Dong X Xu SB Chen X Tao M Tang XY Fang KH. Human cerebral organoids establish subcortical projections in the mouse brain after transplantation. Mol Psychiatry. (2021) 26:2964–76. doi: 10.1038/s41380-020-00910-4 33051604 PMC8505255

[55] Revah O Gore F Kelley KW Andersen J Sakai N Chen X. Maturation and circuit integration of transplanted human cortical organoids. Nature. (2022) 610:319–26. doi: 10.1038/s41586-022-05277-w, PMID: 36224417 PMC9556304

[56] Sharf T Van Der Molen T Glasauer SMK Guzman E Buccino AP Luna G. Functional neuronal circuitry and oscillatory dynamics in human brain organoids. Nat Commun. (2022) 13:4403. doi: 10.1038/s41467-022-32115-4, PMID: 35906223 PMC9338020

[57] Trujillo CA Gao R Negraes PD Gu J Buchanan J Preissl S. Complex oscillatory waves emerging from cortical organoids model early human brain network development. Cell Stem Cell. (2019) 25:558–569.e7. doi: 10.1016/j.stem.2019.08.002, PMID: 31474560 PMC6778040

[58] Fiorenzano A Sozzi E Birtele M Kajtez J Giacomoni J Nilsson F. Single-cell transcriptomics captures features of human midbrain development and dopamine neuron diversity in brain organoids. Nat Commun. (2021) 12:7302. doi: 10.1038/s41467-021-27464-5, PMID: 34911939 PMC8674361

[59] Cakir B Xiang Y Tanaka Y Kural MH Parent M Kang YJ. Engineering of human brain organoids with a functional vascular-like system. Nat Methods. (2019) 16:1169–75. doi: 10.1038/s41592-019-0586-5, PMID: 31591580 PMC6918722

[60] Song L Yuan X Jones Z Griffin K Zhou Y Ma T. Assembly of human stem cell-derived cortical spheroids and vascular spheroids to model 3-d brain-like tissues. Sci Rep. (2019) 9:5977. doi: 10.1038/s41598-019-42439-9, PMID: 30979929 PMC6461701

[61] Sun XY Ju XC Li Y Zeng PM Wu J Zhou YY. Generation of vascularized brain organoids to study neurovascular interactions. elife. (2022) 11:e76707. doi: 10.7554/eLife.76707, PMID: 35506651 PMC9246368

[62] Pham MT Pollock KM Rose MD Cary WA Stewart HR Zhou P. Generation of human vascularized brain organoids. Neuroreport. (2018) 29:588–93. doi: 10.1097/WNR.0000000000001014, PMID: 29570159 PMC6476536

[63] Shi Y Sun L Wang M Liu J Zhong S Li R. Vascularized human cortical organoids (vOrganoids) model cortical development *in vivo*. PLoS Biol. (2020) 18:e3000705. doi: 10.1371/journal.pbio.3000705 32401820 PMC7250475

[64] Qian X Su Y Adam CD Deutschmann AU Pather SR Goldberg EM. Sliced human cortical organoids for modeling distinct cortical layer formation. Cell Stem Cell. (2020) 26:766–781.e9. doi: 10.1016/j.stem.2020.02.002, PMID: 32142682 PMC7366517

[65] Baho E Chattopadhyaya B Lavertu-Jolin M Mazziotti R Awad PN Chehrazi P. p75 neurotrophin receptor activation regulates the timing of the maturation of cortical parvalbumin interneuron connectivity and promotes juvenile-like plasticity in adult visual cortex. J Neurosci. (2019) 39:4489–510. doi: 10.1523/JNEUROSCI.2881-18.2019, PMID: 30936240 PMC6554620

[66] Chattopadhyaya B Di Cristo G Higashiyama H Knott GW Kuhlman SJ Welker E. Experience and activity-dependent maturation of perisomatic Gabaergic innervation in primary visual cortex during a postnatal critical period. J Neurosci. (2004) 24:9598–611. doi: 10.1523/JNEUROSCI.1851-04.2004, PMID: 15509747 PMC6730138

[67] Shi Z Zhang J Chen S Li Y Lei X Qiao H. Conversion of fibroblasts to parvalbumin neurons by one transcription factor, ascl1, and the chemical compound forskolin. J Biol Chem. (2016) 291:13560–70. doi: 10.1074/jbc.M115.709808, PMID: 27137935 PMC4919442

[68] Donegan JJ Tyson JA Branch SY Beckstead MJ Anderson SA Lodge DJ. Stem cell-derived interneuron transplants as a treatment for schizophrenia: preclinical validation in a rodent model. Mol Psychiatry. (2017) 22:1492–501. doi: 10.1038/mp.2016.121, PMID: 27480492 PMC5290293

